# Optimising engagement in a digital parenting intervention to prevent violence against adolescents in Tanzania: protocol for a cluster randomised factorial trial

**DOI:** 10.1186/s12889-023-15989-x

**Published:** 2023-06-23

**Authors:** Roselinde Janowski, Ohad Green, Yulia Shenderovich, David Stern, Lily Clements, Joyce Wamoyi, Mwita Wambura, Jamie M. Lachman, G. J. Melendez-Torres, Frances Gardner, Lauren Baerecke, Esmee Te Winkel, Anna Booij, Orli Setton, Sibongile Tsoanyane, Sussie Mjwara, Laetitia Christine, Abigail Ornellas, Nicole Chetty, Jonathan Klapwijk, Isang Awah, Nyasha Manjengenja, Kudely Sokoine, Sabrina Majikata, Lucie D. Cluver

**Affiliations:** 1 Department of Social Policy and Intervention, University of Oxford, Barnett House, 32-37 Wellington Square, Oxford, Ox1 2ER UK; 2 Centre for Development, Evaluation, Complexity and Implementation in Public Health Improvement (DECIPHer), School of Social Science, Cardiff University, Cardiff, UK; 3 Wolfson Centre for Young People’s Mental Health, Cardiff University, Cardiff, UK; 4 Innovations in Development, Education, and the Mathematical Sciences (IDEMS International), Reading, UK; 5 National Institute for Medical Research Mwanza Research Centre, Mwanza, Tanzania; 6 Centre for Social Science Research, University of Cape Town, Cape Town, South Africa; 7 Parenting for Lifelong Health, Oxford, UK; 8 Faculty of Health and Life Sciences, University of Exeter, Exeter, UK; 9 Clowns Without Borders South Africa, Durban, South Africa; 10 Freelance Designer and Illustrator, Cape Town, South Africa; 11 Innovations in Development, Education, and the Mathematical Sciences (INNODEMS), Kakamega, Kenya; 12 Investing in Children and Strengthening Their Societies, Shinyanga, Tanzania; 13 Department of Psychiatry and Mental Health, University of Cape Town, Cape Town, South Africa

**Keywords:** Parenting, Engagement, Factorial experiment, Optimisation, Low- and middle-income countries, Digital intervention, Multiphase optimisation strategy (MOST)

## Abstract

**Background:**

Violence against adolescents is a universal reality, with severe individual and societal costs. There is a critical need for scalable and effective violence prevention strategies such as parenting programmes, particularly in low- and middle-income countries where rates of maltreatment are highest. Digital interventions may be a scalable and cost-effective alternative to in-person delivery, yet maximising caregiver engagement is a substantial challenge. This trial employs a cluster randomised factorial experiment and a novel mixed-methods analytic approach to assess the effectiveness, cost-effectiveness, and feasibility of intervention components designed to optimise engagement in an open-source parenting app, ParentApp for Teens. The app is based on the evidence-based Parenting for Lifelong Health for Teens programme, developed collaboratively by academic institutions in the Global South and North, the WHO, and UNICEF.

**Methods/design:**

Sixteen neighbourhoods, i.e., clusters, will be randomised to one of eight experimental conditions which consist of any combination of three components (Support: self-guided/moderated WhatsApp groups; App Design: sequential workshops/non-sequential modules; Digital Literacy Training: on/off). The study will be conducted in low-income communities in Tanzania, targeting socioeconomically vulnerable caregivers of adolescents aged 10 to 17 years (16 clusters, 8 conditions, 640 caregivers, 80 per condition). The primary objective of this trial is to estimate the main effects of the three components on engagement. Secondary objectives are to explore the interactions between components, the effects of the components on caregiver behavioural outcomes, moderators and mediators of programme engagement and impact, and the cost-effectiveness of components. The study will also assess enablers and barriers to engagement qualitatively via interviews with a subset of low, medium, and high engaging participants. We will combine quantitative and qualitative data to develop an optimised ParentApp for Teens delivery package.

**Discussion:**

This is the first known cluster randomised factorial trial for the optimisation of engagement in a digital parenting intervention in a low- and middle-income country. Findings will be used to inform the evaluation of the optimised app in a subsequent randomised controlled trial.

**Trial registration:**

Pan African Clinical Trial Registry, PACTR202210657553944. Registered 11 October 2022, https://pactr.samrc.ac.za/TrialDisplay.aspx?TrialID=24051.

**Supplementary Information:**

The online version contains supplementary material available at 10.1186/s12889-023-15989-x.

## Background

Violence against children and adolescents (VAC) is a severe public health issue that disproportionally affects families in low- and middle-income countries (LMICs) [1, 2]. Substantial evidence from large clinical and epidemiological studies (e.g., [3, 4]) and reviews (e.g., [5–8]) has highlighted the extensive short- and long-term adverse consequences for young people, including mental and physical health, education, substance use, and crime. Addressing VAC involves supporting parents and other caregivers to provide safe and nurturing family environments, including through evidence-based parenting interventions [9]. Indeed, research has demonstrated the effectiveness of group-based parenting programmes in reducing VAC, with strong evidence emerging from LMICs [10–12]. Although these programmes typically target parents of young children [11], there is also a growing evidence-base for adolescent-focused parenting interventions in LMICs (e.g., [13, 14]).

Despite the effectiveness of group-based parenting programmes in LMICs, significant access, implementation, and engagement barriers limit their scalability [15]. Contextual and structural factors including geographic restrictions to attending the programme, lack of transport, and parents’ work and caring demands, limit their accessibility and wide-scale reach [15–17]. Access and implementation are further constrained by resource-specific barriers, such as high implementation cost, limited infrastructure for service delivery, and lack of human resources to deliver parenting programmes [11, 12, 15].

In addition to the structural and resource barriers that reduce access to and scalability of evidence-based parenting interventions, other barriers shaped by cultural and social beliefs about parenting [18, 19], family and caregiver factors [20, 21], stigma [22], and intervention delivery formats [23] may also inhibit parental engagement and thus effective scale-up. From a health belief model standpoint, caregivers may not engage with an intervention due to pre-existing attitudes, values, and beliefs [24]. Similarly, the theory of planned behaviour [25] suggests that caregivers’ norms and attitudes as well as the perceived difficulty of carrying out a behaviour, such as attending a parenting programme, influence engagement. Insights from the field of behavioural economics have also been used to understand how contexts, including those shaped by inequality, poverty, and discrimination, can impact engagement with a programme [26].

Collectively, these access, implementation, and engagement barriers highlight the need for carefully designed, culturally appropriate, evidence-based parenting interventions that overcome challenges of accessibility, acceptability, and scalability while being cost-effective.

### Digital interventions as a scalable alternative

With the rapid increase in technology and internet access in LMICs, adaptation of parenting programmes into digital formats has the potential to overcome some of the prominent limitations associated with in-person delivery. Digital parenting programmes, i.e., parenting interventions that use digital technologies to promote behaviour change [27], can be delivered remotely via various platforms including smartphone applications (apps), chatbots, social media, and websites, and range from guided delivery with human support to completely self-guided [28]. These delivery formats may allow for greater scalability, can be accessed irrespective of location and time, and may allow LMICs or underserviced areas to deliver programmes that would otherwise be too costly.

A small but growing number of digital parenting programmes have been tested in high- and middle-income countries to date, with meta-analytic and systematic reviews from high-income countries indicating that they may yield effects comparable to in-person parenting interventions (e.g., [28–33]). However, a major concern that prevents their successful implementation is low engagement by parents. For example, only 7.5% of parents who enrolled in a self-guided online parenting programme in Australia completed all core modules and post-intervention assessments [34]. Looking at the digital intervention literature more broadly, approximately 48% of participants drop out of smartphone mental health intervention trials before the end of data collection [35]. Retention rates are estimated to be even lower in real-world settings. For example, a systematic review investigating real-world uptake and engagement of digital self-help interventions found that while 21% to 88% of participants used the intervention at least once or completed one assessment or module; only 0.5% to 28.6% completed the intervention [36].

As a precursor to behaviour change, poor engagement in parenting programmes may compromise intervention effectiveness [37]. Consequently, low engagement has significant negative implications for population-level public health benefit. Therefore, optimising engagement strategies for digital parenting programmes is crucial to realise their full preventative potential and address the widespread issue of VAC in LMICs.

### Optimising engagement

Engagement with digital interventions has been variably defined depending on the specific technology or context of interest. An integrative definition following a systematic review of 117 qualitative and quantitative studies on engagement in digital behaviour change interventions proposed that engagement comprises 1) “the extent (e.g., amount, frequency, duration, depth) of usage” and 2) “a subjective experience characterised by attention, interest, and affect” [37]. This definition views engagement as a multidimensional process that is influenced by factors linked to the user, intervention features, and socio-contextual influences [37, 38]. For instance, user factors shown to enhance engagement with some digital interventions include less severe family baseline symptoms, positive perceptions about the programme and the targeted behaviour, previous positive experiences with the technology or platform, and fewer perceived risks and consequences of using the intervention [39, 40]. The literature has also identified intervention-specific factors that are associated with increased engagement in digital interventions, including peer-based social networking features such as forums and group chats, tailored and personalised content, and support from trained professionals [39, 41]. Yet research studies which have rigorously evaluated the relationship between intervention-specific components and engagement in digital parenting interventions remain largely absent.

One approach to systematically developing, optimising, and evaluating multiple intervention components simultaneously is the Multiphase Optimisation Strategy (MOST; [42]). MOST involves three phases: Preparation, Optimisation, and Evaluation. In the Preparation Phase, researchers develop a conceptual model using theory and literature to identify potential components hypothesised to impact outcomes of interest (e.g., engagement, cost, effectiveness), conduct formative work and feasibility piloting, and define the optimisation objective, i.e., how effectiveness will be balanced against affordability, scalability, and efficiency [43]. The Optimisation Phase rigorously assesses the performance of the intervention components identified in the Preparation Phase, enabling the selection of effective and the rejection of ineffective components. This can be achieved using several experimental designs, including randomised factorial experiments, which, unlike traditional randomised controlled trials (RCTs), allow for the evaluation of multiple intervention components and their interactions within one trial [44]. The results from the optimisation trial, together with the optimisation objective, are then used to select the optimised intervention package. Lastly, the Evaluation Phase is aimed at evaluating the optimised intervention in a traditional two-armed RCT. MOST has been used to optimise engagement and effectiveness in an in-person parenting programme in LMICs [45], and in a range of digital public health interventions in high- and middle-income countries including adult depression [46], smoking cessation [47], HIV pre-exposure prophylaxis [48], and adult weigh loss [49]. However, to our knowledge, MOST has yet to be applied to the optimisation of engagement in digital parenting interventions.

### Objectives

Guided by the MOST framework, this study aims to identify the most effective, cost-effective and feasible/acceptable engagement package for a recently adapted app-based parenting intervention, ParentApp for Teens (henceforth referred to as ParentApp) targeting vulnerable caregivers of adolescents ages 10 to 17 years in Tanzania. The Preparation Phase of MOST, including a formative user testing and feasibility pilot of ParentApp, occurred between 2020 and 2022. The current paper describes the study protocol for the Optimisation Phase of MOST. The protocol follows the SPIRIT guidelines for clinical trials [50], and the completed checklist is reported in Additional file 1.

#### Primary objectives

1. To identify which of the selected component levels contribute meaningfully to improvements in the primary engagement outcomes (overall number of app-launches, proportion of workshops/modules completed, proportion of home practice activities started).
2. To identify which of the selected component levels contribute meaningfully to improvements in the secondary engagement outcomes (overall time spent on app, proportion of workshops/modules started, number of ParentPoints logged).

#### Secondary objectives

3. To conduct exploratory analyses to examine whether there are any interaction effects between components on primary and secondary engagement outcomes.
4. To conduct exploratory analyses of the potential impact of components and combination of component levels on caregiver behavioural outcomes (e.g., child maltreatment, positive parenting, parental communication about sexual abuse prevention).
5. To conduct exploratory analyses to examine whether caregiver baseline characteristics (e.g., gender, age, financial stress and food insecurity, caregiver stress and depression, and parenting behaviour) are potential moderators of component effectiveness.
6. To estimate the incremental cost-effectiveness of component levels delivered during programme implementation.
7. To qualitatively understand participant perspectives on the feasibility and acceptability of the component levels and the overall challenges and enablers of engaging with ParentApp.
8. To identify the most effective, cost-effective, and feasible/acceptable combination of component levels to be tested further in an RCT in 2023.

## Methods/design

### Overview of the study

We will use a 2 × 2 × 2 cluster randomised, multifactorial experiment to examine the effectiveness and cost-effectiveness of three intervention components designed to enhance engagement with ParentApp. The intervention components are: A) Support (self-guided, moderated WhatsApp groups), B) App design (sequential workshops, non-sequential modules), and C) Digital literacy training (on, off) (see Table 1). Each of these components was selected based on learnings from the Preparation Phase and addresses one or more theoretical mediator associated with poor engagement in digital parenting interventions (described in detail below).

**Table 1:** Experimental conditions for a 2 × 2 × 2 factorial trial (*N* = 16 clusters, two clusters per experimental condition, 80 participants per condition)

Group	Component A (Support)	Component B (App Design)	Component C (Digital Literacy)	*n*
1	Self-guided	Non-sequential	On	80
2	Self-guided	Non-sequential	Off	80
3	Self-guided	Sequential	On	80
4	Self-guided	Sequential	Off	80
5	WhatsApp	Non-sequential	On	80
6	WhatsApp	Non-sequential	Off	80
7	WhatsApp	Sequential	On	80
8	WhatsApp	Sequential	Off	80

Component A: “Self-guided” refers to use of the app without any external support; “WhatsApp” refers to use of the app with additional moderated WhatsApp group support; Component B: “Non-sequential” refers to the app version where content is presented in a non-sequential task-based modular format; “Sequential” refers to the app version where content is presented in a time-bound weekly format; Component C: “On” refers to receiving a short, structured digital literacy training at the group onboarding session; “Off” refers to not receiving the digital literacy training

To account for contamination and potential confusion that could arise if participants learn about the different experimental conditions, random assignment to conditions will be clustered. We will randomly allocate 16 low-income neighbourhoods in Mwanza City, Tanzania, to eight different treatment combinations (*N* = 16 clusters, approximately 40 caregivers in each cluster, approximately 640 caregivers in total). Two clusters will be allocated to each experimental condition with approximately 80 families per condition. Although this factorial trial has eight different combinations of the three intervention components, it is important to note that each component will be evaluated by comparing all caregivers receiving one component to all caregivers not receiving that component. Compared to a traditional RCT, this design is highly efficient as it enables the testing of several components simultaneously, requiring a smaller sample to test their effects [42].

In addition, this study will also assess the barriers and facilitators to engagement qualitatively via interviews and focus groups discussions (FGDs) with a subset of low, medium, and high engaging participants (*N* = 30). This qualitative approach allows for the investigation of caregivers’ subjective engagement with the intervention, providing a nuanced understanding of their perceptions about the feasibility and acceptability of intervention components and the contextual factors influencing engagement. Using the component selection methods outline by Collins and colleagues [51] in conjunction with qualitative findings, we will identify an intervention package for ParentApp which is optimally effective, cost-effective, and feasible for low-income families in Tanzania. The optimised intervention package will subsequently be evaluated in a two-arm RCT.

### Intervention

ParentApp is a smartphone app adapted from the in-person, group-based parenting programme called Parenting for Lifelong Health (PLH) for Teens. PLH for Teens was developed and tested in South Africa through a collaboration between academic institutions, the World Health Organization (WHO), UNICEF, and local non-government organisations and is one of the few parenting programmes that have been developed for and tested with families in LMICs [13]. Since 2012, PLH for Teens has been delivered in 18 countries to over 250,000 families [52], including 40,000 families in Tanzania [53].

ParentApp is open source, non-commercialised, and developed for offline use. It targets families with adolescents aged 10 to 17 and aims to prevent violence against adolescents, support parenting, and reduce risks of sexual violence exposure. Content and delivery were jointly developed by the universities of Oxford, Cape Town, and Tanzania’s National Institute for Medical Research (NIMR), in collaboration with Innovation in Development, Education, and Mathematical Sciences (IDEMS International and INNODEMS Kenya), Investing in Children and Strengthening their Societies (ICS), and Clowns Without Borders South Africa (CWBSA).

Content is delivered through text, images, and audio in 12 interactive workshops or modules which mirror the in-person programme in a condensed format. Core content is combined with reminders to relax, suggestions for family activities, messages of praise, and scheduled push-notification reminders to complete activities. After completion of each workshop/module, caregivers practice the skills they have learnt with their families in home-practice activities. Other core features of the app include a habit-tracking tool, ParentPoints, where caregivers log activities and positive parenting behaviours, and a library that provides instant access to resources such as essential parenting tips and activities, information on local support, and technical support. Caregivers can also choose whether to participate alone or in a group.

During the Preparation Phase, we conducted surface adaptations from the in-person PLH for Teens programme implemented in Tanzania [53], including condensing content from 14 to 12 sessions and using culturally appropriate illustrations that were gender-neutral, amorphous cartoon figures. Subsequent stages included iterative user testing with caregivers and adolescents from nine LMICs [54]. Results from the user testing demonstrated high satisfaction and acceptability of content. However, participants’ views on the app’s animated cartoons varied, with some finding them too juvenile. Participants also highlighted the need for regular reminders to encourage engagement. Following feedback from the user testing, we conducted a pilot study of intervention feasibility when delivered as a self-guided programme in low-income communities in South Africa. Findings from the pilot with 107 caregivers and nine implementing staff support those from the user testing; that the content is acceptable, relevant, and useful, and that the app is easy to use. Nevertheless, participant engagement in the pilot was low, with as little as 25% of recruited participants still engaging after the first session. Technical challenges and internet access were cited as some of the main difficulties for participants. Other feedback included wanting flexible access to content, and an alternative interface with human images. Participant feedback also suggested the need for an element of regular human support. Several adaptations were made following these findings and are being piloted in Tanzania, including testing the feasibility of remote support via one-on-one phone consultations and moderated WhatsApp groups.

**Fig. 1:**
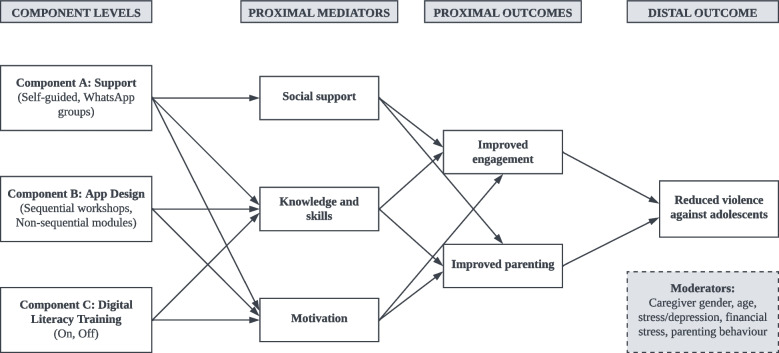
Conceptual model of ParentApp for Teens

Findings from the Preparation Phase as well as the larger digital intervention literature guided the selection of intervention components to be tested in the multifactorial experiment (see conceptual model in Fig. 1). The following criteria were used for component selection: each component must address at least one theoretical mediator associated with engagement with digital parenting interventions; each component must be unique from other components in terms of delivery mode, approach, or content; each component must contain at least some initial evidence in the literature; and each component and component level must be relatively low-cost and feasible for implementation in low-resource settings.

### Description of intervention components

The following three components will be evaluated in the factorial experiment: A) Support, B) App Design, and C) Digital Literacy Training. If needed, we may modify the components prior to study implementation in response to unforeseen changes to our operational capacity or the findings from the ongoing pilot in Tanzania.

#### Component A: support (self-guided/WhatsApp groups)

Research has shown that engagement and impact of digital interventions are significantly influenced by the degree of guidance provided during the programme [41]. Guided interventions integrate human support (typically from clinicians, peers, or lay workers) via digital platforms, such as messaging, phone calls, and video conferencing. Self-guided interventions do not provide any support from a person but may include automated messaging and reminders. Although self-guided interventions tend to be more scalable and cost-effective, current reviews indicate that digital interventions without guidance experience lower engagement rates and reduced effects compared to those that are guided (e.g., [39, 55–57]). Improved engagement and outcomes in guided interventions may be mediated by the therapeutic/working alliance and social support that professionals or peers provide [38]. However, what is unclear is the amount and format of guidance that is necessary for enhanced engagement in digital interventions.

Feedback from facilitators, who conducted WhatsApp groups and individual phone consultations with participants in the Tanzanian pilot, suggests that WhatsApp groups may be a more efficient and feasible mode of support compared to phone consultations in the Tanzanian context. Thus, this study will test two levels of support, 1) participants receive no external support and use ParentApp as a self-guided intervention, or 2) participants receive ParentApp alongside additional support via WhatsApp groups of approximately 40 participants per group, moderated by a local trained facilitator.

#### Component B: app design (sequential/non-sequential)

In-person parenting programmes, including PLH for Teens, typically deliver sessions in a sequential, weekly basis. ParentApp was originally designed to mirror this structure, i.e., content is delivered in a sequential order, whereby a new workshop is made available once every programme week. However, qualitative findings from the pilot study in South Africa indicated that some participants wanted to access content more flexibly and at their own pace. In addition, findings from this pilot suggested that participants typically used the app in multiple, short sessions. This suggests that a short, task-based approach may be an acceptable mode of delivery. Previous studies of digital parenting programmes have used both sequential access (e.g., [58, 59]) and open access methods (e.g., [60]). However, none have investigated which approach is more effective at engaging and retaining participants.

To better understand the impact that sequential versus non-sequential access to content has on engagement, we will test two versions of the app: 1) a workshop-based design where participants access content in a sequential and timed-bound manner, and 2) a module-based design where content is broken down into smaller tasks and participants can access content in a non-sequential manner. Thus, participants will be randomised across two component levels: 1) sequential workshop-based design and 2) non-sequential modular design. Based on participant feedback in the Preparation Phase, these two designs also differ visually: the sequential design will retain the amorphous cartoon figures and the non-sequential design will incorporate human-like illustrations.

#### Component C: digital literacy training (on/off)

A wide range of implementation and service-level strategies are routinely used to support engagement in parenting programmes. These include the provision of childcare, transport or financial aid, meals, and, seldomly, rewarding families with small gifts for attendance [61]. However, for digital parenting programmes, particularly in low-income settings where access to technology is not as widespread, digital literacy may be a key barrier to engaging with the intervention. This was highlighted in the Preparation Phase of this study, where technical challenges related to digital literacy, phone compatibility, network connectivity, and data access were frequent, particularly in the initial set-up phase. A recent systematic review investigating barriers and facilitators to engagement in 208 digital mental health interventions found that participants’ experience of technical challenges was a primary barrier to engagement, whereas digital literacy and previous positive experiences with the technology influenced the extent to which users engaged with the intervention; with higher digital literacy being associated with greater engagement [39]. The review also found that interventions that provided training on how to use the technology or the intervention achieved higher engagement. Digital literacy training may therefore be a critical implementation strategy to support engagement with digital interventions. This study will test the effectiveness of a digital literacy training as a strategy to maximise engagement with the app by randomising participants across two component levels: 1) receiving a brief, semi-structured digital literacy training at the group onboarding session, i.e., where participants are screened for eligibility, provide informed consent, and install the app on their phones, and 2) receiving no digital literacy training at the group on boarding session.

### Hypotheses

1. For the support component, we hypothesise that participants who receive WhatsApp groups will show higher levels of engagement compared to participants not receiving facilitated support.
2. For the app design component, we hypothesise that participants who receive non-sequential module design will show higher levels of engagement compared to participants who receive sequential workshops.
3. For the digital literacy component, we hypothesise that participants who receive the digital literacy training will show higher levels of engagement compared to participants receiving no digital literacy training.

### Study setting

The study will be conducted in low-income areas of Mwanza City, a port city next to Lake Victoria in Northern Tanzania. The study site was selected in consultation with research and implementation partners in Tanzania, the Tanzanian National Institute for Medical Research (NIMR) and local NGO Investing in Children and Strengthening their Societies (ICS).

### Participants and eligibility criteria

Recruitment will target socioeconomically vulnerable families from low-income communities, situated in peri-urban and urban regions of Mwanza City. Eligible clusters will be low-income neighbourhoods with appropriate sites for conducting onboarding sessions with approximately 40 participants. Examples of potential sites are community halls, schools, and community leaders’ offices. Eligible caregivers are: 1) age 18 or older, 2) the main caregiver of a teenager aged 10 to 17 years, 3) live in the same household as the target teenager for at least four nights per week in the previous month, 4) have regular access to an android smartphone, and 5) provide written informed consent. If a participant is unable to read or has a severe learning disability that affects their ability to give informed consent, they will not be included for ethical reasons.

For the collection of qualitative data, targeted sampling will be used to identify participants for participation in individual interviews or FGDs (approximately *N* = 30). Participants will be selected based on their level of engagement: low engagement (completing ≤ 4 workshops or modules, *n* = 10); moderate engagement (completing ≥ 5 and ≤ 8 workshops or modules, *n* = 10); high engagement (completing ≥ 9 workshops or modules, *n* = 10).

### Participant timeline

**Fig. 2:**
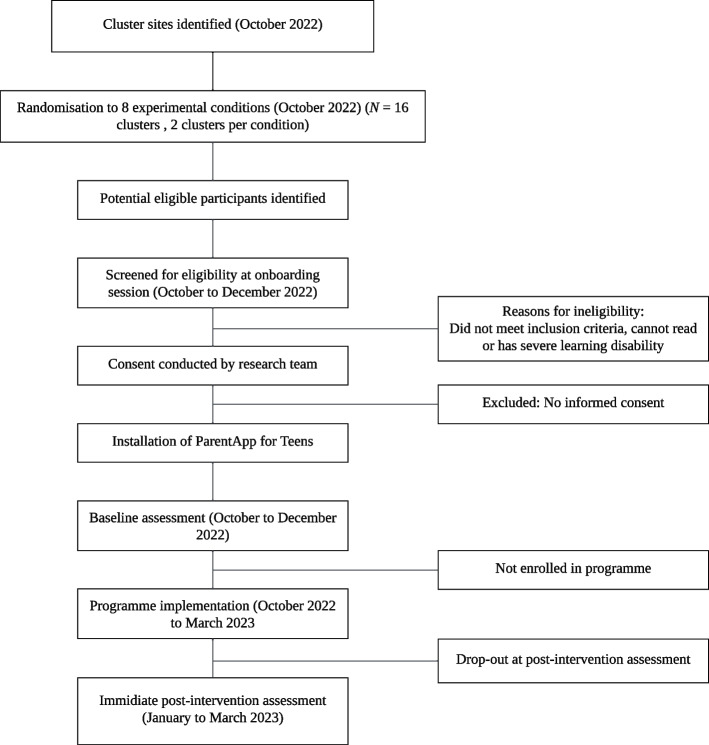
Study flow diagram

The participant timeline is shown in the study diagram (Fig. 2). Recruitment will rely on our in-country partners, NIMR and ICS, to work with local leaders to identify eligible caregivers. Recruitment pathways of caregivers will be through existing NGO services, schools, and/or individual families needing support. Quota sampling, a non-probabilistic version of stratified sampling [62], will be used to ensure that there is an adequate representation of male caregivers in the sample (approximately 20% males will be recruited per cluster).

Potential caregivers will be invited to attend an in-person onboarding session where they will be screened for eligibility. Recruitment will continue until full study enrolment of an estimated 40 caregivers is achieved per cluster (October to December 2022). If necessary, recruitment will include peer-to-peer referrals in the community. At onboarding sessions, participants will provide informed consent and receive assistance to install and download the app on their smartphones from trained research assistants. Those who provide informed consent will be considered enrolled. They will then complete the self-administered embedded baseline assessment with support from the trained research assistants. At the end of the onboarding session, digital literacy training will be provided to participants in select clusters. Participants receiving moderated WhatsApp groups will be added to a group within the first week of enrolment. Post-assessments will be completed by participants on their smartphones approximately three months after baseline (January to March 2023) without the presence of a trained research assistant.

### Randomisation and blinding

Cluster randomisation of neighbourhoods (*N* = 16) will involve a single-stage randomisation processes assigning each neighbourhood, i.e., cluster, to one of 8 experimental conditions [63]. Local research managers will send a list of the selected neighbourhoods to a member of the off-site research team prior to randomisation. Random allocation of clusters to experimental conditions will then be conducted using a randomisation algorithm in R. After randomisation, a list of the cluster allocation to experimental conditions will be sent back to the in-country research team. The specific combination of component levels received by caregivers will depend on the onboarding session they attend which is nested within the neighbourhood they were recruited form. Blinding will not be possible for facilitators and local research staff due to their involvement in programme implementation. However, participants will not be informed about the range of possible conditions. The data analyst will be blinded from condition assignment during analysis.

### Measures and outcomes

All quantitative engagement data are derived from the app itself, which tracks app usage throughout the intervention. Socio-demographic and caregiver behavioural measures are derived from a self-administered pre-post questionnaire imbedded within the app. The questionnaire was designed with a dual aim of a) tailoring content to the needs of caregivers (for example using their name, and highlighting workshops/modules that may be particularly important to them) and b) assessing preliminary effectiveness of the programme without being too burdensome and assuming minimal digital literacy. Thus, it only includes items related to the core outcomes ParentApp targets and the most basic socio-demographic information. Items were selected based on a subset of measures previously used in the in-person PLH for Teens implementation in Tanzania [53]. All items originate from open-access measures that have been psychometrically validated in previous studies, including in LMICs. To further ensure that the items were culturally relevant and acceptable in the specific context of our study, we engaged in a co-development process with Tanzanian experts on VAC, conducted iterative pre-piloting with Tanzanian parents, and pilot-tested the measures with 100 Tanzanian families, including both female and male caregivers and their adolescents. We also conducted in-depth qualitative interviews with a subgroup of these families. Where possible, item response scales were standardised to a frequency scale (0 to 8 or more times) to make reading and comprehension easier for participants. Study assessments are summarised in Table 2.

**Table 2:**
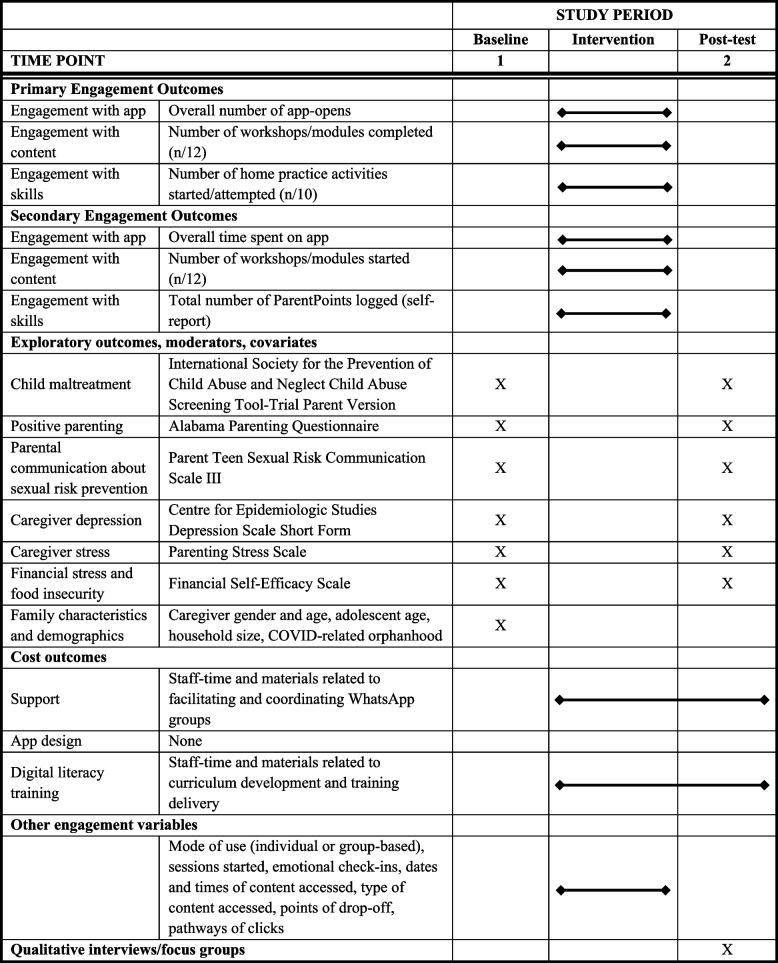
Summary of study assessments

#### Engagement measures

Engagement in digital interventions can be viewed as a three-phase process that is non-linear in nature [64]. In the first phase, participants explore the digital platform to decide if it is useful and relevant. In the second phase, participants use part of the intervention or content. In the third phase, participants return to engage with the content and activities more fully. In this study, we will capture these three phases, while concurrently measuring the amount, frequency, duration, and depth of usage as defined by Perski and colleagues [37]. After reviewing literature, consulting experts, and drawing from experience with engagement and attendance in in-person parenting interventions, the three phases were operationalised as engagement with the app, content, and skills/activities. For each phase, a primary engagement variable was chosen based on its potential impact on parenting practices, while a secondary engagement outcome was selected to assess less commonly measured aspects of engagement. By measuring engagement in this way, we hope to gain insight into how participants engage with the intervention and which variables are most important for achieving desired effects.

##### Primary engagement outcomes (throughout intervention period)

We will assess three primary outcomes, one from each of the three engagement domains outlined above. *Engagement with the app* will be defined as the overall number of app-launches throughout the intervention. *Engagement with the content* will be based on the proportion of workshops or modules completed (out of 12). *Engagement with the skills* will be defined as the proportion of home practice activities started or attempted (out of 10).

##### Secondary engagement outcomes (throughout intervention period)

We will also assess three secondary outcomes, each based on one of the three engagement domains outlined above. *Engagement with the app* will be defined as the overall time spent on the app throughout the intervention. *Engagement with the content* will be the defined as the proportion of workshops or modules started (out of 12). *Engagement with the skills* will be defined as the total number of ParentPoints (self-reported habit tracker) logged throughout the intervention.

##### Other engagement measures

For exploratory and descriptive purposes, additional usage measures may include mode of app use (individual-based or in a group with other caregivers), number of workshops/modules started but not completed, responses to emotional check-ins, dates and times of content accessed, type of content accessed, points of drop-off, and pathways of clicks/navigation. Additionally, engagement with the embedded baseline and endline assessments will be assessed and treated as an outcome of the study. These findings, including retention and dropout rates will be used to inform the forthcoming RCT following the Optimisation Phase of ParentApp.

#### Moderators, co-variates and exploratory outcomes

##### Socio-demographic measures (co-variates, moderators, sample description)

Caregivers will provide information about themselves and their family in the imbedded questionnaire. This will include caregiver gender and age, adolescent age, household size, and one item assessing COVID-related orphanhood (“has a caregiver of your teen passed away in the past three years”). Financial stress will also be assessed using two items adapted from the Financial Self-Efficacy Scale [65]. One item asks, “how many times in the past month have you felt very worried or anxious about money?”. Caregivers respond on a frequency score, on a scale of 0 to 8 or more times. The second item assesses food insecurity “how many days (out of 30) did you run out of money to pay for food?”.

##### Child maltreatment (exploratory outcome, moderator)

Four items adapted from the reduced version of the ISPCAN Child Abuse Screening Tool-Trial Parent Version [66] will be used to assess child maltreatment. The tool asks caregivers to report the frequency of emotional abuse (2 items, “shout, scream or yell” and “insult or call names or stupid”) and physical abuse (2 items, “hitting, spanking or slapping with a hand or with an object like a stick or belt” and “discipline with a push or grab”) over the past month using a scale of 0 to 8 or more times. Items sum to create a score for each subscale as well as a total child maltreatment score.

##### Positive parenting (exploratory outcome, moderator)

Five items from the Alabama Parenting Questionnaire [67] will be used to assess positive parenting. Caregivers will be asked to give a frequency score of their behaviours towards their adolescents in the past month on a scale of 0 to 8 or more times. The tool measures positive parenting (1 item, “how many times have you praised your teen?”), involved parenting (1 item, “get involved in activities that your teen likes”, and parental supervision (3 items, e.g., “stay out in the evening past the time they are supposed to be home”). Items sum to create a total positive parenting score as well as a score for the parental supervision subscale.

##### Parental communication about sexual risk prevention (exploratory outcome, moderator)

Three items adapted from the Parent Teen Sexual Risk Communication Scale III [68] will be used to assess adolescent sexual risk behaviour and caregiver communication about sexual risk prevention. One item asks, “how many times in the past month has your teen walked home alone, taken a lift with someone they don’t know or hung out in a place that made you worried for their safety?”. Caregivers respond on a frequency scale ranging from 0 to 8 or more times. Risk scenarios were adapted based on high-risk scenarios in Tanzania, in discussion with local researchers and based on available evidence. The other item asks, “have you ever talked with your teen about ways to avoid being touched or made to do sexual things with people or online?”. Caregivers respond yes or no. If they respond yes, they will be asked “how many times in the past month have you had a talk like this?”. Caregivers are asked to respond on a frequency ranging from 0 to 8 or more times.

##### Parental depression (moderator)

Three items from the Centre for Epidemiologic Studies Depression Scale (CES-D 10) [69] will be used to assess caregiver depression. The items ask caregivers to respond how they have felt in the previous week (3 items, e.g., “felt that everything you did was an effort”). Caregivers respond on a frequency scale ranging from 0 to 7 days. Items are summed with higher scores indicating greater parental depression.

##### Parenting stress (moderator)

One item adapted from the Parenting Stress Scale [70] will be used to assess caregiver stress. The item asks, “how many times in the past month did caring for your children make you feel very stressed” The items will be rated on a frequency scale of 0 to 8 or more times.

#### Cost outcomes

Costs related to two of the three intervention components will be considered in this study. These include all costs associated with delivering the support component of the intervention, as well as the digital literacy training component. These costs will be collected by the relevant implementing staff using cost diaries. Costs will include money and time spent on programme delivery, i.e. materials and staff-time required for training, preparation, delivery, and coordination of activities. Costs associated with the development of the app are being excluded, since real-world deployments will effectively happen at zero-marginal cost, excluding the cost associated with content adaption. This is because the app has been developed to be completely self-contained, such that no additional supporting infrastructure is needed for routine rollout. In addition, costs incurred by participants (e.g., time spent using the app), and the evaluation of the intervention will be excluded from analyses, as the focus of this study is to understand scale-up cost, feasibility and effectiveness.

### Data collection

Data collected will include digitally tracked engagement data, embedded questionnaire questions, and semi-structured qualitative interviews or FGDs. Questionnaire data includes sociodemographic and pre-post caregiver behavioural questions which are integrated into the first and the last ParentApp workshops/modules. Participants will complete the baseline assessment at the onboarding session, where they can seek support from research assistants. Research assistants have received extensive training from study investigators on interviewing techniques, ethics, and informed consent. All measures will be translated from English to Kiswahili and back translated. Cost data will be collected by research assistants, programme facilitators, coaches, and field coordinators throughout the study.

Interviews and FGDs will be conducted by local researchers with a subsample of participants following semi-structured interview guides. The interview guides are designed to facilitate discussion but remain flexible, allowing for the inclusion of emerging themes during data collection. Interviews and FGDs will be audio-recorded in addition to researchers taking backup notes. Before recording, permission will be sought from all participants, and in situations where the participants decline, notes will be taken. In cases where in-person interviews and FGDs are not possible, remote interviewing via telephone will take place.

Collecting digital engagement data in this trial requires participants to access the internet regularly so that their app activity data can sync with the online server. To improve the quality and accuracy of the data collected, we will provide participants with small monthly data bundles (approximately 1 GB). Additionally, to promote participant retention, participants who complete the questionnaire and semi-structured interview or FGDs will receive compensation for their time (approximately $2 per assessment).

### Data management and confidentiality

ParentApp is used offline with usage data stored on the device. When participants activate their data bundles, usage data and other in-app data will be automatically uploaded through end-to-end encryption to an IDEMS International server. Data will then be de-identified and shared with dedicated members of the research team by being uploaded to a secure server at the University of Oxford. Electronic records from remote data collection and in-person onboarding procedures will be exported from the respective platforms to a secure server at the NIMR, Tanzania, or the University of Oxford. This includes electronic records of audio recordings, transcripts, screening and consent forms, and participant tracking lists. Non-electronic data will be stored in locked filing cabinets at our local partners’ offices in Tanzania. Confidentiality will be maintained by delinking all personal identification in the final datasets used for analysis. All anonymised data will be kept for five years, in accordance with the University of Oxford’s ethical standards and all non-anonymised data will be disposed of after study completion.

### Data analysis

Quantitative data will be cleaned and analysed in R. Analyses will be conducted on an intention-to-treat basis, i.e., participants who have dropped out after enrolment will be included in the analysis. However, if there is significant dropout, we will exploratively analyse causal effects using complier average effects of components [71]. Patterns of missingness will be inspected, applying multiple imputation with fully conditional specification where appropriate [72].

#### Primary and secondary outcomes (Objective 1 and 2)

Generalised linear mixed effects modelling will be used to examine the main effect of each component level (e.g., those receiving sequential workshops versus those receiving non-sequential modules) on primary and secondary engagement outcomes. This approach includes a random intercept for clusters which will account for nesting of participants in neighbourhoods. The appropriate error distribution and link function will be selected for each outcome variable (e.g., overall app launches will be parametrised using a Poisson distribution; proportion of workshops completed will be parameterised using a Binomial distribution; proportion of home practice activities will be parameterised using a Binomial distribution). Main effects will be modelled as fixed effects and evaluated at the 0.05 significance level along with 95% confidence intervals. To assist the interpretation of effects, effect coding (where component levels take on the value of -1 or 1) may be applied [51, 74]. The regression will also include baseline covariates centred on the sample mean including caregiver gender and age, adolescent age, financial stress, and parenting behaviour such as child maltreatment.

#### Statistical power and sample size considerations for primary objectives

Given our focus on participant engagement and the intricate nature of the study design, we opted for a simulation-based approach to ascertain the appropriate sample size and statistical power [75]. We generated data for a 16-cluster randomised experiment with eight unique experimental conditions, varying the number of participants within each cluster (mean of 40, standard deviation of 8). We built the analysis model outlined above for each of the primary outcomes (i.e., overall app launches, proportion of sessions completed, and proportion of home practice activities started), estimating engagement rates and model parameters based on existing digital parenting intervention research (e.g., [32]), digital mental health interventions implemented in real-world settings (e.g., [36]), the original PLH for Teens cluster RCT in South Africa [13], and plausible scenarios based on observations from the ParentApp feasibility pilot. For each outcome model, 10,000 datasets were generated.

We estimated the minimum effect sizes needed to achieve ≥ 80% power for each of the primary outcomes while taking into account clustering and assuming an alpha level of 0.05. Poisson and Binomial distribution models were fitted for count and factor outcomes, respectively. Effect sizes were calculated as incidence rate ratios (IRRs) for Poisson distribution models and odds ratios (ORs) for Binomial distributions, which were averaged across all 10,000 replications. For overall app launches, the mean IRRs were 1.14 for support (WhatsApp), 0.91 for app design (sequential workshops), and 1.21 for digital literacy (on). For the proportion of sessions completed, the mean ORs were 2.18 for support (WhatsApp), 0.48 for app design (sequential workshops), and 2.35 for digital literacy (on). For the proportion of home practice activities started, the mean ORs were 3.13 for support (WhatsApp), 0.67 for app design (sequential workshops), and 2.31 for digital literacy (on). Based on these results, we determined that a sample of 640 participants would be sufficient to detect a significant effect in each of the primary outcomes.

#### Exploratory analyses

##### Objective 3: To conduct exploratory analyses to examine whether there are any interaction effects between components on primary and secondary engagement outcomes

We anticipate that there will be some interactions (synergistic effects) between component levels. In exploratory analyses, we will examine a fully saturated generalised linear mixed effects model which includes all three main component effects, as well as two-way and three-way interactions. This model will be replicated for each of the primary and secondary outcomes. However, interaction effects will not be considered in the final component level selection process due to potential power constraints.

##### Objective 4: To conduct exploratory analyses of the potential impact of components and combination of component levels on caregiver behavioural outcomes (e.g., child maltreatment, positive parenting, parental communication about sexual abuse prevention)

Engagement is a precursor to behaviour change. We will therefore investigate whether experimental components designed to enhance engagement also impact caregiver behavioural outcomes at post-intervention. Using the same generalised linear mixed effects models outlined above, we will examine the main and interaction effects of intervention components on the core ParentApp targets: reducing child maltreatment, improving positive parenting and supervision, and increasing parental communication about sexual abuse prevention. Covariates will additionally include baseline assessments of each outcome. We may also conduct exploratory mediation analyses to generate learning around which engagement variables mediate programme impacts.

##### Objective 5: To conduct exploratory analyses to examine whether caregiver baseline characteristics (e.g., gender, age, financial stress and food insecurity, caregiver stress and depression, and parenting behaviour) are potential moderators of component effectiveness

In exploratory analyses, we will test the potential moderation of each component by socio-demographic information such as caregiver gender and age, as well as baseline severity of caregiver stress, depression, financial stress, and parenting behaviours. If appropriate, stratified analysis or effect modification will be used, adding interaction terms for the treatment component and the potential moderator [76] and considering separating effect modification into contextual and person-level effects.

#### Cost-effectiveness (Objective 6)

The study will follow the two-stage cost-effectiveness analysis procedure described in Bernstein, Dziura [77]. In the first step, we will build an incremental cost-effectiveness table where the total cost associated with each of the 8 experimental conditions will be compared against the average number of workshops completed within those conditions. Conditions that are clearly dominated in terms of cost to engagement ratio will be dropped, without regard to the statistical significance. In the subsequent more refined analysis step, the marginal cost of delivering each level of the constituent intervention components will be compared to the marginal effectiveness of the component; effectiveness will once more be defined in terms of number of workshops completed. The reason for considering this primary outcome in particular is that unlike the outcome associated with the higher form of engagement (that is, engagement with the skills, i.e. home-practice activities), it is not a self-reported measure. Incremental cost-effectiveness ratios will be estimated alongside 95% confidence intervals.

#### Qualitative analysis (Objective 7)

A novel feature of this study is the use of qualitative interviews to assess facilitators of and barriers to engagement. The aim of this approach is to ensure that intervention components are considered acceptable and feasible by participants. Qualitative interviews will be audio-recorded, transcribed, and then translated into English. Researchers will independently review a sample of transcripts and draw on the research questions and early engagement findings to generate a coding framework. The data will then be jointly reviewed to reach a consensus on the coding scheme. Thereafter, researchers will analyse the data thematically by identifying overarching themes, relationships, and concepts [78]. Data will be analysed with the support of QSR-NVivo qualitative analysis software.

#### Analytic strategy for selecting final intervention package (Objective 8)

**Fig. 3:**
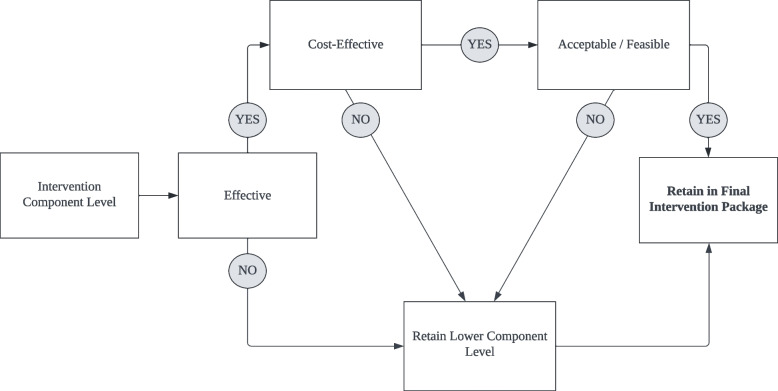
Analytic strategy for selecting final component levels for subsequent RCT

The optimisation criteria for this study are to select the most effective, cost-effective, and feasible component levels for inclusion in the optimised intervention. Using the decision-making framework outlined by Collins and colleagues [51], we will apply the following selection procedures. First, we will examine the main effects to determine whether a component has an effect overall, averaged across the other two components. If a significant differential effect is detected, we will tentatively select the effective component level. If a component has no main effects or a negative effect, we will tentatively retain the lower component level or the level hypothesised to perform worse. If a component level has a main effect, its cost-effectiveness will then be examined. We will then draw on qualitative findings to examine the acceptability and feasibility of the retained component level. Component levels found to be acceptable and feasible in the interviews and FGDs with participants (that are also effective and cost-effective) will be included in the final optimised intervention (Fig. 3).

### Oversight and monitoring

#### Trial oversight

A Trial Steering Committee (TSC) has been assembled and will meet quarterly to ensure that the study is conducted with rigour. The trial coordinating team, composed of the Principal Investigators, trial managers, and key members from our partner organisations will meet regularly to oversee the management of research activities.

#### Data monitoring

Given that this is a low-risk intervention (there were no safety concerns in the ParentApp pilot study or any of the PLH effectiveness trials), no external data monitoring committee will be formed. Data safety and monitoring will be undertaken by the trial coordinating team. However, should the TSC deem it necessary, a data monitoring committee will be assembled during the study who will have access to interim analyses and make any final decisions regarding trial termination.

#### Ethical considerations and consequences of participation

##### Ethical issues regarding staff

All staff have prior experience working on community research projects in similar communities to our study. Nevertheless, conducting research in vulnerable communities can be mentally challenging and potentially harmful. As such, all research assistants will be trained in awareness and safety measures, and will not be required to undertake assessments in situations where they feel uncomfortable or unsafe. Additionally, staff will travel in pairs when working in areas that may be less safe.

Moreover, we recognise that research with vulnerable families can be emotionally challenging due to the disclosure of sensitive information. We will conduct weekly debriefing meetings with research personnel to discuss any potentially distressing events that may have occurred during data collection.

##### Potential of harm to participation

This study is committed to upholding the universal principles of human research ethics (respect, beneficence, and justice) throughout all stages of the trial. As the focus of the study is to enhance caregiver engagement in a digital parenting programme for socioeconomically vulnerable families, we believe that the potential benefits, such as reducing the risk of child maltreatment and improving family well-being, outweigh any potential harm to participants. Nevertheless, we acknowledge the possibility of harm at two levels: participation in the study and participation in the intervention.

##### Potential harm from the study

Participants may experience emotional distress brought on from disclosing challenging emotions and experiences during interviews, FGDs, and when filling in the self-administered outcome questionnaires. All interviewers will be trained and experienced in working with vulnerable families. Our research team also includes a Tanzanian community health specialist, Dr. Wamoyi, who is skilled in child and adolescent health, public health, and community health. Dr. Wamoyi will be available to discuss or supervise discussion of any issues that may arise with the participants following the interviews. If there is a need for a participant to access more extensive support (such as seeing a counsellor or attending a clinic), we will provide direct referrals to relevant service providers through partnerships with local organisations. Additionally, direct referrals to social services will also be available through the implementing partner and intervention facilitators, should risk of harm become apparent during the group-based onboarding session or any other stage of the study.

All research personnel, including research assistants and facilitators, will be trained in ethical procedures and protocols concerning research with human subjects. During the consent stage, we will inform all participants that everything said will be confidential unless it becomes clear that the participant and/or a family member are at risk of harm. We will also inform participants that they have the right to refuse to answer any questions that they feel uncomfortable with and may stop the interview or withdraw from the study at any time without any negative consequences.

##### Disclosure of harm or potential harm

Participants may also disclose experiences of ongoing violent practices, whether as the recipient or the perpetrator of harm toward themselves, their child(ren) and/or partner that may require intervention. This study recognises that researchers have a responsibility towards safeguarding children and other individuals who may be at risk of experiencing abuse or neglect, or any other forms of severe harm. The following protocol is proposed to mitigate any actual or potential harm to children or adults that might occur during the study.

If it is disclosed that the participant (who will be aged 18 or older) has recently experienced inter-personal violence (physical, sexual, or emotional) or is at risk of experiencing inter-personal violence, and he or she would like a direct referral to a health, welfare or other services, the data collector or relevant member of the research team will abide by the following:

1. If information in face-to-face data collection is disclosed that suggests that any member of the household is at risk of significant harm, the researcher will discuss concerns with the respondent at the end of the interview. In the self-administered outcome assessments, the referral process will be triggered based on participant survey responses that indicate potential harmful experiences and/or support needs, including experiences of violence, mental health issues, substance abuse, neglect, and food insecurity. In such cases, the participant will be contacted via phone call by a member of the research team.
2. If the individual at risk is the participant, a member of the research team will inform the respondent that he/she may choose to self-refer (by contacting a service provider on the list of services given to the participant, or another provider of his/her own choosing) or request a direct referral to health, welfare or other services.
3. If the participant requests a direct referral, the member of the research team will complete an adult referral form together with the participant.
4. If the participant is in immediate danger, or his or her own emotional state is such that it places their own or a third party’s life at risk, the member of the research team will urgently share the information with the appropriate service provider (e.g. police, ambulatory service, nearest hospital).
5. If the case does not appear to be acute, the research team member will agree upon a timeframe with the participant when the information will be shared with the appropriate service provider.
6. If the individual at risk of harm is a child, the researcher will discuss with the parent/caregiver the options for referral to child welfare, health organisations, and other services.
7. If the harm towards the child is considered significant, the research member will inform local child protection services immediately and the participant will be automatically excluded from the study. If authorities do not respond immediately, the researchers will follow up until assistance arrives.
8. The research team member responsible for the referral will work with the in-country project coordinator to monitor whether the service provider has indeed responded to the request for support. Weekly supervision meetings with all field personnel will allow discussion of issues that arise concerning harm to research subjects and children.
9. If we determine that respondents or their families have experienced significant harm as a result of participation in the study (i.e., severe abuse, suicidality, intimate partner violence, or other potential psychological or physical injuries), further research activities will be suspended until the issue has been adequately addressed and the study has been adapted accordingly.

##### Mitigating potential harm from intervention

We have also considered the potential risk of harm from participating in the intervention, and will be monitoring this throughout the project [79]. Participation in the parenting programme may potentially cause psychological harm for caregivers, particularly when they confront difficult experiences from their own childhood or deal with intimate partner violence at home. However, past research on parenting interventions, including numerous randomised trials conducted in LMICs [11], have consistently shown no evidence of harm and abundant evidence of benefits for both parents and children, as well as high levels of parent satisfaction. To ensure a comprehensive understanding of the potential outcomes of the parenting programme, this study will explicitly examine both the benefits and risks of the intervention. Our statistical analysis plan includes two-tailed tests to assess the differences between groups and examine potential positive and negative effects of the intervention. We will also use qualitative analysis to explore the experiences and perspectives of participants regarding the intervention and its effects.

We anticipate no direct harm as a result of withdrawing from the intervention, as participation in the programme is completely voluntary, with no direct penalties for non-participation. Furthermore, other studies, including evaluations in other low-resource settings [11], have shown no evidence of harm resulting from the termination of parenting interventions. In the unlikely event of significant harm being observed in any of the intervention conditions, we will suspend the implementation of the programme until the harm has been adequately addressed, and the programme has been modified accordingly.

##### Self-Referral

In addition to our referral procedure described above, the app includes local self-referral and emergency contacts which are affordable and remote-friendly. This information will include services for family and child support, substance use, gender-based violence and rape, child abuse and protection, physical, mental, and contact details for available helplines. These resources were identified by the research team following a thorough mapping of online and affordable support systems in Tanzania.

### Dissemination plans

Findings will be shared with policy makers, government stakeholders, community-based organisations, and community members. Results will also be published in open-access peer-reviewed journals and presented at conferences. Authorship of publications from this study will follow the guidelines recommended by the International Committee of Medical Journal Editors. All components of this research, including the research protocol, study assessments, anonymised datasets, and statistical code will be made available on the Open Science Framework.

In addition to academic dissemination and policy engagement, community dissemination and participant feedback will be prioritised. Brief reports in lay language summarising the study findings will be created, focusing on the findings relevant to NGOs and government working with caregivers and adolescents. These reports will be presented to local community groups, NGOs, and health services. Researchers will also report back verbally to participants, encouraging their thoughts and feedback on emerging findings. For example, the later stages of the qualitative research will ask participants to respond to some of the quantitative findings, which will serve as a form of feedback. No identifiable details will be given in any dissemination or feedback.

## Discussion

This trial is the first of its kind to optimise engagement strategies for a digital parenting intervention in a LMIC. It involves a pragmatic cluster randomised factorial experiment and an innovative mixed-methods analytic approach to inform the selection of the most effective, cost-effective and feasible engagement package for ParentApp, implemented among vulnerable caregivers of adolescents in Tanzania. By examining the role of human support, app design, and a digital literacy training in reducing barriers to programme participation, this study promises to provide key insights into engagement and effectiveness whilst tailoring to vulnerable population needs, delivery challenges, and best-practises for scale-up. Furthermore, findings may also identify potential moderators that could provide understanding about effective engagement strategies for different subpopulations such as fathers. Thus, this novel trial has the potential to inform pragmatic implementation strategies for digital interventions, specifically as they relate to engaging and retaining vulnerable families in digital public health prevention services in LMICs.

## Protocol amendments

The current protocol version is 1.0 (24/10/2022). Any significant subsequent protocol modifications will be submitted to the study investigators and the Institutional Review Boards for approval.

## Supplementary Information

**Additional file 1.** SPIRIT Checklist.

**Additional file 2.** ParentApp information sheet and consent form for caregivers.

## Data Availability

ParentApp for Teens is freely available and open source online. Anonymised datasets will be made available via an open-access repository such as the Open Science Framework (https://osf.io/).

## Abbreviations

VAC: Violence against children
LMICs: Low- and middle-income countries
NGO: Non-governmental organisation
MOST: Multiphase optimisation strategy
RCT: Randomised controlled trial
PLH: Parenting for Lifelong Health
NIMR: National Institute for Medical Research in Tanzania
ICS: Investing in Children and Strengthening their Societies
FGDs: Focus group discussions
IDEMS International: Innovations in Development, Education, and the Mathematical Sciences
INNODEMS Kenya: Innovations in Development, Education, and the Mathematical Sciences
IRR: Incidence rate ratio
OR: Odds ratio
TSC: Trial Steering Committee
