# Supplementary material for: Optimising engagement in a digital parenting intervention to prevent violence against adolescents in Tanzania: protocol for a cluster randomised factorial trial
Source: BMC Public Health. 2023 Jun 23;23:1224. doi: 10.1186/s12889-023-15989-x (PMC10288745; doi:10.1186/s12889-023-15989-x)
Supplement: Supplementary file 2 — Additional file 2. ParentApp information sheet and consent form for caregivers. [file 12889_2023_15989_MOESM2_ESM.docx]

##
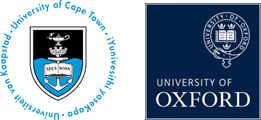
Additional File 2: Information Sheet and Consent for Participation in the Optimisation Study with Facilitated WhatsApp Group Support


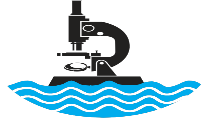

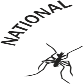

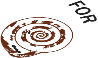


**CAREGIVER PARTICIPANT INFORMATION SHEET - PARENTAPP WITH WHATSAPP**

You are invited to take part in a collaborative study conducted by researchers from the National Institute for Medical Research (NIMR), University of Cape Town in South Africa and the University of Oxford in the United Kingdom. Before you decide whether you would like to be part of this study, it is important for you to understand why the research is being done and what it will involve.

**Why is this research being conducted?**

We are doing this study because we would like to test our parenting app, ParentApp for Teens.  We are interested in how you use the app so that we can better understand how to improve it for families with adolescents. We are also interested in changes in your family, your relationships, and parenting before and after participating in the programme.

**Why have I been invited to take part?**

You have been invited to take part in this study because you are a parent of an adolescent aged 10-17 and have regular access to an android smartphone. In order to participate, you will need to provide consent to confirm you are willing to participate in the study.

**Do I have to take part?**

No, your participation is completely voluntary. This means that you do not have to participate if you do not want to. Even if you do agree to take part, you can stop taking part in the research at any time without any consequences or loss. If you want to withdraw, please let one of the research team members know; you do not need to give any reason, and there will be no penalty. Once you have let the research team know that you would like to withdraw, we will stop processing and delete your personal information from the study.

**What will happen to me if I take part in the research?**

If you agree to take part in this study, you will be given a chance to ask any questions that you have about the study and be asked to sign the consent form. Next, a trained ParentApp facilitator will help you download and install the app on your phone. You will also receive WhatsApp group support from a trained facilitator for one to two hours a week.

With your permission, some personal information about you and your family, as well as how you use the app will be directly collected through the app, which will be shared with us for research purposes like understanding how the app functions and your experience with the app.

Your participation will last for about 12 weeks.

**Expenses and payments**

We will cover any internet data costs associated with your participation, for example, data for using the app and for participating in the WhatsApp group.  The amount of data you will receive will be around 1GB per month for the duration of 4 months.  You will also receive a small gift of appreciation for answering some questions in the app at the end of the programme (valued at around TZs 4500).

**Time commitment**

Taking part in the ParentApp programme will take around 8 - 10 hours across 12 weeks (30 – 45 minutes per app module) Taking part in the WhatsApp group will take around 1-2 hours per week.

**Are there any benefits in taking part?**

The information you provide will help us to ensure ParentApp makes a real difference for families in your community and other communities across Africa.

**Are there any potential risks in taking part?**

We do not expect any risks in participating in this study.  All information you give us will be kept confidential. If any questions make you feel uncomfortable, you don’t have to answer them and there will be no penalty against you for this decision.

If you become distressed or find anything in the programme and would like to talk to someone afterwards, we can help to provide you with contact details where you can get support services. You can also find contact details directly in the app for local support services. Remember, you may at any point stop participating whenever you want to without giving a reason.

**What will happen to the information I provide?**

The personal information that we collect only includes what is necessary for the study. The information you share with us (e.g., your consent form in-app information and usage) will be kept safe on a secure cloud server and backed up on servers at the University of Oxford and at the University of Cape Town. It may be transferred to other countries so that we can share it with our research partners involved in similar research. Our research partners include the National Institute of Medical Research Tanzania, the University of Cape Town, the University of Oxford, the University of the Western Cape, IDEMS International, and INNODEMs, Investing in Children and Strengthening their Societies (ICS), the World Health Organisation and UNICEF, but over time more research partners will join this research team and may have access to the data.

We will keep your identifying details separately and only restricted study staff will have access. The rest of the information will be entered into another database, identified only by your study number, and we will only use this database to find the answers to our study questions. This means all information will remain confidential and private. All the data will be stored for five years after the study, but the data that allows you to be identified (e.g., your name) will be destroyed at the end of the study. We may contact you in the future to ask whether you would like to be interviewed about your experiences of participating in the programme. We may also contact you to ask if you would like to be part of longer-term research about this programme.

Study information may be reviewed by ethics committees, and independent monitors, to check that the study procedures were done correctly, and the information is correct. Your information will remain confidential unless we are required by law to release information.

When the study is finished, we will make information from the study available to be shared with other researchers. This will only be done after all the information which identifies people who took part has been removed, so the identities of the people who took part will remain confidential. You have the right to request access to your personal information at any time or request that we correct or destroy any information that you have provided before it is fully anonymised.

**What will happen to the results of the research?**

The results from the study will be presented to people working in the field of parenting programmes, to other researchers and to governments and other agencies in the form of presentations, publications in academic journals and policy briefs. In all these presentations and reports, it will not be possible to identify people who took part.

**Data protection**

The National Institute for Medical research, the University of Oxford and University of Cape Town are responsible for ensuring the safe and proper use of any personal information you provide. We will only process data for research purposes. Research is a task that is performed in the public interest.

**Who has reviewed this study?**

This study has been approved by the National Institute for Medical research (approval number NIMR/HQ/R.8a/Vol.IX/3856), the University of Cape Town Department of Psychology’s Research Ethics Committee (PSY2020-001), and the University of Oxford Ethics Committee (R69744/RE001).

**Who do I contact if I have a question, a concern, or a complaint about the study?**

Please let us know if you have any questions about participating in the study before providing consent. Should you have any questions, concerns, or would like to lodge a complaint about the study, please ask the research team who gave you this form or contact Dr Joyce Wamoyi at the National Institute for Medical Research. The number is 0282500399.

If you have any further questions or concerns about your rights as a study participant, you can contact: Dr. Safari Kinunghi, director at the National Institute for Medical Research. He can be contacted by telephone on 0282500399 and by e-mail at kinunghi_csm@hotmail.com.

The National Health Research Ethics Sub-Committee (*NatHREC*), National Institute for Medical Research has reviewed this study. If you have any questions or concerns about your rights as a person taking part in a research study, or if you wish to make a complaint about the study, you may contact:

Secretary of the National Institute for Medical Research Ethics Committee

National Institute for Medical Research,

3 Barack Obama Drive,

11101 Dar es salaam,

Tanzania.

Landline: +255 22 2121400

Mobile: +255 758 587885

Email: ethics@nimr.or.tz/ nimrethics@gmail.com

Thank you!

**Informed Consent to Take Part in the ParentApp Optimisation Study**

|  | **Please mark** |
| --- | --- |
| 1. *I confirm that I have read and understand the information sheet for the above study.  I have had the opportunity to think about the information, ask questions and have had these answered to my satisfaction.* |  |
| 1. *I understand that my participation in this study is voluntary and that I am free to withdraw at any time, without giving any reason.* |  |
| 1. *I understand that by signing this form I am agreeing to my information being collected.* |  |
| 1. *I understand that all information gathered from this study will be strictly confidential unless required by law.* |  |
| 1. *I understand that sections of data collected during the study may be looked at by responsible individuals from the research partners listed on the information sheet.* |  |
| 1. *I agree to being included in the facilitated WhatsApp group.* |  |
| 1. *I understand who will have access to my data and how it will be stored, shared and published.* |  |
| 1. *I understand how to make a complaint or raise any concerns about my participation.* |  |
| 1. *I am aware that I can request my data be updated or removed at any time before it has been anonymised.* |  |
| 1. *I agree to take part in this study.* |  |
| 1. *I agree to the researcher(s) contacting me after the study has been completed, to ask if I would like to be part of longer-term research or participate in an interview about this programme.* |  |

_____________________________________________ ____________

Printed Name of Parent Taking Part in Study Date

_____________________________________________

Signature of Parent Taking Part in Study

**Statement of Person Obtaining Informed Consent**

I have carefully explained to the person taking part in the study what he or she can expect from their participation. I confirm that this research subject speaks the language that was used to explain this research and is receiving an informed consent form in their primary language. This research subject has provided legally effective informed consent.

_____________________________________________                             ____________

Signature of Person Obtaining Informed Consent                            Date

_____________________________________________

Printed Name of Person Obtaining Informed Consent
